# Is PPR Epidemiological Research Addressing Global Eradication Priorities? A Scoping Review (2015–2025)

**DOI:** 10.3390/v18080857

**Published:** 2026-08-05

**Authors:** Gezahegn Alemayehu, Theodore Knight-Jones

**Affiliations:** Health Program, International Livestock Research Institute, Addis Ababa P.O. Box 5689, Ethiopia; t.knight-jones@cgiar.org

**Keywords:** peste des petits ruminants, epidemiology, scoping review, GREN, surveillance, molecular epidemiology, transmission dynamics, vaccination epidemiology

## Abstract

The PPR Global Research and Expertise Network (GREN) plays a central role in identifying research priorities supporting the Global Peste des Petits Ruminants (PPR) Eradication Programme (GEP). A scoping review of PPR epidemiological research published between 2015 and 2025 was conducted to assess progress and remaining gaps across research domains relevant to GREN priorities. A total of 670 PPR-related publications were retrieved. Epidemiological research constituted 46.9% of the PPR literature, pathogenesis (19.1%), and diagnostics (15.6%), followed by vaccinology (9.5%) and immunology (7.9%). From the 273 epidemiological studies, most originated from Africa (53.1%) and Asia (37.7%). The epidemiological studies were categorized into seven domains. Molecular epidemiology and virus strain characterization represented the largest proportion of published studies (40.0%), followed by surveillance and disease monitoring (23.8%), transmission dynamics and risk modelling (12.5%), vaccination epidemiology (10.6%), analytical and risk factor epidemiology (8.4%), control strategy evaluation (2.9%), and socioeconomic research (1.8%). Progress towards GREN research priorities was variable. Molecular epidemiology and surveillance demonstrated the greatest methodological advancement, supported by widespread application of phylogenetics, serological surveillance, and expanding sequencing capacity across endemic settings. Transmission dynamics and risk modelling also showed increasing analytical sophistication through spatial modelling, network analysis, ecological suitability modelling, and dynamic simulation approaches, although applications remained geographically concentrated and only partially integrated into operational decision-support systems. In contrast, vaccination epidemiology, comparative evaluation of control strategies, and socioeconomic research remained limited in volume and operational integration. Across domains, important gaps persisted in implementation-focused evaluation, integrated surveillance systems, and translation of research evidence into adaptive eradication planning and policy development. Overall, the findings demonstrate substantial growth in the global PPR epidemiological evidence base over the past decade but also reveal a persistent imbalance across research domains and geographies. While major advances have been achieved in molecular characterization and descriptive surveillance, greater integration of epidemiological, operational, modelling, and socioeconomic evidence is needed to strengthen adaptive eradication planning and improve alignment between research priorities and policy implementation. Research and policy need to be better integrated, with policy needs driving research focus and research findings then informing policy. Experience from rinderpest eradication demonstrates that strong research–policy interfaces are essential to ensure that scientific evidence is translated into effective eradication strategies and decision-making.

## 1. Introduction

Peste des petits ruminants (PPR) is a highly contagious viral disease of sheep and goats that imposes a substantial global burden on small ruminant production systems. The disease causes annual global economic losses estimated at USD 1.45–2.1 billion, driven by high mortality (up to 90% in naïve populations), reduced productivity, trade disruptions, and control costs [1]. Approximately 80% of the world’s 2–3 billion small ruminants are at risk across nearly 70 endemic countries in Africa, the Middle East, and Asia, with an additional 50 countries considered vulnerable to incursion. The impacts fall disproportionately on an estimated 300 million pastoralists and smallholders whose livelihoods, food security, and nutrition depend heavily on small ruminants [2].

Beyond direct herd losses, PPR outbreaks trigger cascading socioeconomic effects, including market instability and export restrictions. The burden is particularly pronounced in sub-Saharan Africa and South Asia, where small ruminants contribute substantially to household income and agricultural GDP [1,3,4].

In response, the Food and Agriculture Organization and the World Organization for Animal Health launched the PPR Global Control and Eradication Strategy (PPR-GCES) in 2015, establishing a strategic framework and a four-stage progressive pathway to achieve global eradication of PPR by 2030 [5]. Building on this strategy, the PPR Global Eradication Programme (PPR-GEP) was subsequently developed as the operational framework to support implementation through coordinated surveillance, vaccination, capacity strengthening, monitoring, and evaluation across endemic countries [2]. Supported by the PPR Global Research and Expertise Network (GREN), the PPR-GEP strategy builds on lessons from rinderpest eradication and frames PPR elimination as a global public good [2].

As PPR eradication progresses, existing and new challenges need to be addressed. GREN advises PPR GEP on research findings that can help address these challenges and identifies priority research areas to advance global PPR control. As part of a wider GREN initiative, we review progress in PPR research since the inception of PPR GEP in 2015, focusing on epidemiology and assessing progress in priority research areas identified by GREN. In so doing, we identify critical knowledge gaps that may constrain progress towards global eradication.

## 2. Methods

### 2.1. Review Design

The review methodology followed the framework established by the Joanna Briggs Institute (JBI) for scoping reviews [6] and was reported in accordance with the Preferred Reporting Items for Systematic Reviews and Meta-Analysis (PRISMA) Extension for Scoping Reviews (PRISMA-ScR) [7]. The completed PRISMA-ScR checklist is included in the Supplementary Materials (Table S1). Consistent with JBI guidance for scoping reviews, a formal risk-of-bias assessment was not conducted because the objective of the scoping review was to map the breadth of epidemiological evidence, identify research patterns and thematic distribution, and detect evidence gaps in relation to eradication priorities rather than effect estimation. No protocol registration was conducted.

### 2.2. Review Questions

This scoping review was guided by the Population–Concept–Context (PCC) framework.

- Population: Studies involving small ruminants (sheep, goats) and wild ruminant species susceptible to peste des petits ruminants (PPR) in mixed livestock–wildlife systems, where PPR epidemiology was explicitly addressed.
- Concept: Epidemiological research including surveillance, molecular epidemiology, transmission dynamics and risk modelling, vaccination epidemiology, socioeconomic impacts, and evaluation of control strategies.
- Context: All PPR research published from 2015 to 2025, considering research needs for global eradication.

Primary review question: What is the scope, distribution, and extent of epidemiological research published since the start of the PPR Global Eradication Program in 2015?

### 2.3. Eligibility Criteria

Studies were eligible for inclusion if they were published between 1 January 2015 and 31 December 2025 and reported primary epidemiological research. This includes, but is not limited to, surveillance and monitoring studies, risk factor analyses, transmission dynamics and modelling studies, socioeconomic studies, vaccination epidemiology, and evaluations of prevention and control strategies. Only full-text articles published in peer-reviewed journals were considered. Systematic reviews were included only if they offered substantial analytical synthesis that provided new epidemiological insights beyond merely descriptive summaries. The literature search was not restricted by language at the identification stage, and studies published in languages other than English were captured where English titles and/or abstracts were available. However, only studies for which full-text screening and standardized data extraction could be conducted in English were included in the final synthesis due to feasibility and resource constraints related to translation.

Studies were excluded if they met any of the following criteria: They did not focus on PPR or fell outside the field of epidemiology. This includes laboratory-based studies such as diagnostic assay development, immunology, pathogenesis, and/or vaccine development that lack explicit epidemiological application or population-level analysis. Additionally, non-original research publications, such as opinion pieces, editorials, commentaries, operational manuals, narrative reviews, and conference abstracts, were also excluded.

### 2.4. Search Strategy

A structured search was conducted in PubMed using a combination of Medical Subject Headings (MeSH) and free-text terms related to PPR. The search strategy was designed to maximize sensitivity and capture all relevant PPR publications. PubMed was selected as the primary database because it captures the majority of indexed PPR epidemiology literature; however, the authors acknowledge that additional databases may have identified further studies.

The search was limited to publications from 1 January 2015 to 31 December 2025. The complete PubMed search string used was as follows: (“Peste-des-Petits-Ruminants” [Mesh] OR “peste des petits ruminants” [Title/Abstract] OR “PPR virus” [Title/Abstract] OR “PPRV” [Title/Abstract]) AND (“1 January 2015” [Date-Publication]: “31 December 2025” [Date-Publication]).

### 2.5. Study Selection

All identified records were imported into Rayyan (www.rayyan.ai), accessed on 1 November 2023, an online review management platform, where duplicate entries were detected and removed before screening. The study selection process was conducted in two sequential stages: (1) title and abstract screening and (2) full-text eligibility assessment.

**Figure 1:**
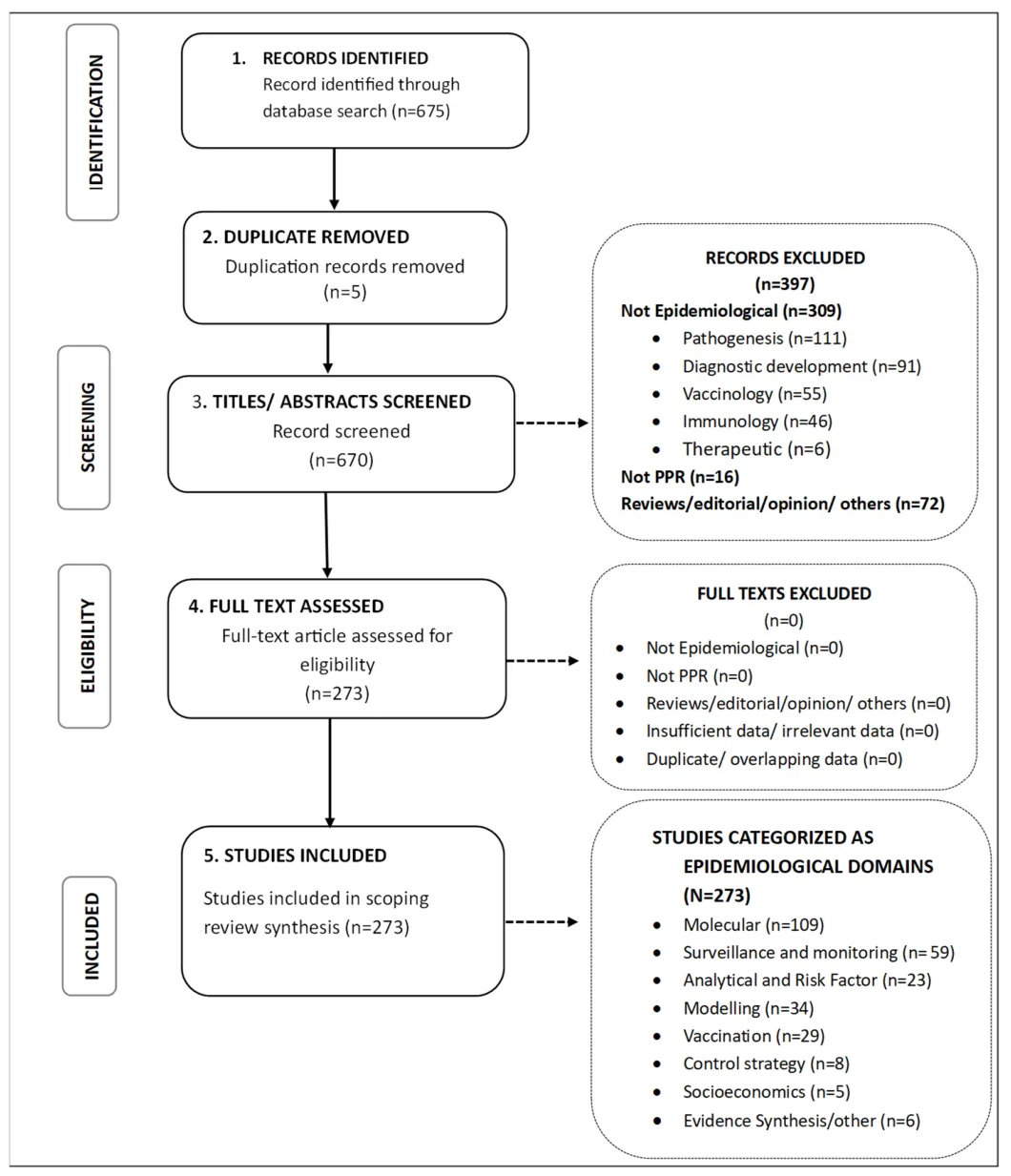
PRISMA flow diagram for the study selection process, adapted from the PRISMA 2020 template [8] in accordance with PRISMA-ScR guidelines [7]. Arrows indicate the sequential progression of records through the review process.

During the first stage, two reviewers (GA and TJK) independently screened titles and abstracts to identify potentially relevant publications related to PPR. Studies were provisionally classified into predefined broad research domains (e.g., epidemiology, pathogenesis, diagnostics, vaccinology, immunology, therapeutics, and other relevant categories) based on information available in the title and abstract. This review was intentionally limited to epidemiological research priorities, consistent with its objective of synthesizing evidence on progress in epidemiological research supporting the PPR Global Eradication Programme (PPR-GEP). Accordingly, studies focusing primarily on vaccine development, diagnostic assay development, immunology, pathogenesis, therapeutics, and other non-epidemiological research domains were considered outside the predefined scope of this review. Given the focus on epidemiology, full-text articles were retrieved and assessed for papers within the epidemiology domain. Publications categorized as non-epidemiological were retained for research domain categorization but were not subjected to a detailed full-text eligibility assessment or data extraction. The selection process is illustrated in Figure 1 (PRISMA flow diagram).

### 2.6. Identification of Priority Research Areas

To interpret how the evolving epidemiological evidence base aligns with the operational needs of the PPR GEP, this review was structured according to research priority areas identified by the PPR-GREN. PPR-GREN is a technical advisory platform established in 2018 to support evidence generation and guide research priorities relevant to progressive PPR control and eradication, jointly coordinated by the FAO and the WOAH [9,10].

Priority research areas were identified through a review of the seven PPR-GREN meeting reports [10,11,12,13,14,15,16]. Key themes identified included molecular epidemiology and virus characterization, surveillance and disease monitoring, transmission dynamics and risk modelling, vaccination epidemiology, analytical and risk factor epidemiology, socioeconomic dimensions of disease control, wildlife and atypical host epidemiology, and evaluation of control and eradication strategies [11].

These priority areas served as a framework for the categorization of the included studies. During data extraction and synthesis, studies were grouped into epidemiological domains according to their primary research focus, methodological orientation, and relevance to operational eradication priorities aligned with PPR-GREN thematic working groups [16].

### 2.7. Data Extraction and Charting

Data extraction was conducted using a standardized charting form developed a priori to ensure consistency and reproducibility. Studies were categorized into different types using a predefined framework (Table 1). This classification framework was developed iteratively based on GREN thematic priorities and refined during the pilot extraction of the included studies. Each study was assigned one primary domain based on its central research objective and dominant methodological approach. Some papers covered more than one epidemiology domain.

One reviewer performed the initial extraction, and a second reviewer independently verified the data for accuracy. Discrepancies were resolved through discussion and consensus. For each study included, the following variables were extracted: study identifier, year of publication, geographic region, primary epidemiological domain, secondary epidemiological domains (if applicable), host type, diagnostic method(s), and analytical method(s). The complete list of included studies and extracted study characteristics is provided in the Supplementary Materials (Table S2).

### 2.8. Data Synthesis

A descriptive quantitative synthesis was conducted to summarize the distribution of studies across epidemiological domains. Frequencies and proportions were calculated based on the primary domain classification. Domain distributions were further stratified by publication year, geographic region, host type, and methodological approach. Temporal trends were assessed descriptively to identify shifts in research emphasis over the study period (2015–2025). Geographic breadth was assessed based on the regional dispersion of studies across endemic zones, using an evidence-mapping matrix (Region × Domain).

A qualitative assessment was then done to assess the progress level of the domains, considering the range and sophistication of methods applied, geographic breadth (from localized to multi-country coverage), and policy/control relevance (from limited academic insight to direct influence on vaccination campaigns or control programs). The level of progress was defined as follows: ‘Advanced’ denotes a mature field with sophisticated methods, broad geographic representation, and clear policy linkages; ‘Intermediate’ indicates solid methodological foundations but significant constraints in breadth or policy translation; ‘Emerging’ reflects an underdeveloped field with basic methods, limited scope, and minimal policy impact.

## 3. Results

### 3.1. Result of Study Selection Process

From 2015 to 2025, a total of 675 publications on PPR were identified (Figure 1). After removing duplicates (*n* = 5), 670 unique records were screened based on their titles and abstracts. During title and abstract screening, 397 (59.3%) records were excluded. These included reviews/editorials/opinion papers (*n* = 72), studies not related to PPR (*n* = 16), and non-epidemiological publications: pathogenesis studies (*n* = 111), diagnostic assay studies without epidemiological application (*n* = 91), vaccine development studies without field or population-level evaluation (*n* = 55), immunology studies (*n* = 46), and therapeutic studies (*n* = 6).

A total of 273 (40.7%) studies met the inclusion criteria and were included in the final scoping review synthesis. The included studies were distributed across epidemiological domains as follows: molecular epidemiology (*n* = 109), surveillance and disease monitoring (*n* = 59), analytical and risk factor epidemiology (*n* = 23), modelling and spatial epidemiology (*n* = 34), vaccination epidemiology (*n* = 29), control strategy studies (*n* = 8), socioeconomics (*n* = 5), and evidence synthesis (*n* = 6).

### 3.2. Overall PPR Research Landscape

**Figure 2:**
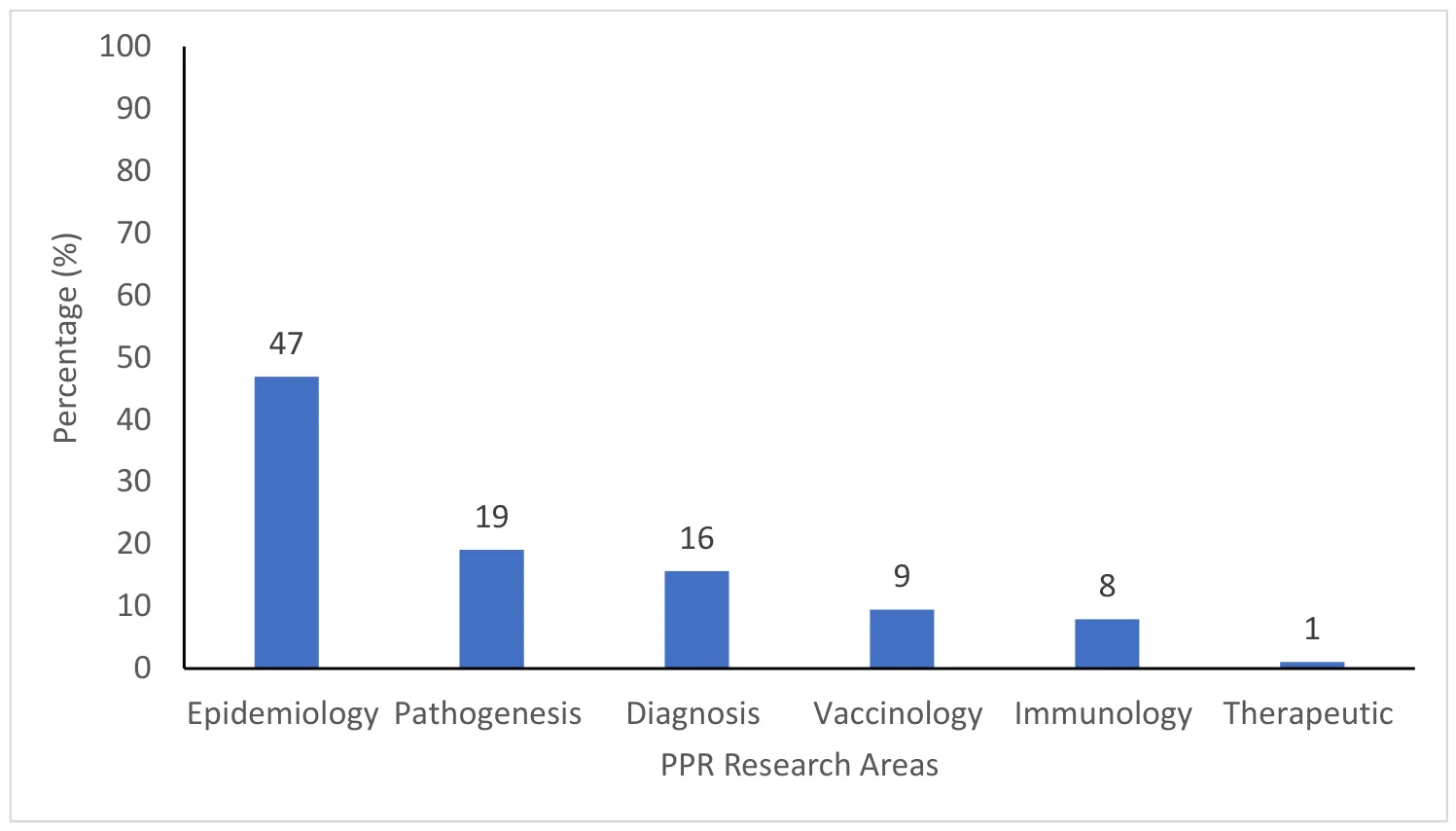
Categorization of PPR research (2015–2025).

Figure 2 illustrates the distribution of published PPR research from 2015 to 2025. Epidemiology constituted the largest proportion of studies published (46.9%), followed by pathogenesis (19.1%), diagnostics (15.6%), vaccinology (9.5%), and immunology (7.9%), while therapeutic research accounted for only 1.0%.

### 3.3. General Characteristics of the Included Studies

A total of 273 studies published between 2015 and 2025 were included (Table 2). Most studies were conducted in Africa (53.1%) and Asia (37.7%), with fewer from Europe (5.5%) or global reports (3.7%). The majority focused on domestic animals (94.5%), with a small proportion on wildlife (5.5%). Within the domain of epidemiology, molecular epidemiology dominated the literature (40%), followed by surveillance and disease monitoring studies (21.6%). Modelling and spatial analyses accounted for 12.5%, while vaccination epidemiology represented 10.6%. Analytical and risk factor studies comprised 8.4%. In contrast, control strategy evaluation (2.9%), evidence synthesis (2.2%), and socioeconomics (1.8%) were minimally represented.

Many studies utilized laboratory-based diagnostic approaches, with PCR and sequencing methods featured in 94 studies (34.4%), conducting molecular characterization and viral lineage analysis. Serological methods were also prevalent, appearing in 83 studies (30.4%) and used to assess seroprevalence and immunity. Secondary data analyses and database-driven studies comprised 48 publications (17.6%), while social survey methods were used in 28 studies (10.3%). Only 17 studies (6.2%) employed mixed molecular and serological methods, and purely clinical assessments were rare, with just 3 studies (1.1%).

#### 3.3.1. Temporal Trends by Epidemiological Domain (2015–2025)

**Figure 3:**
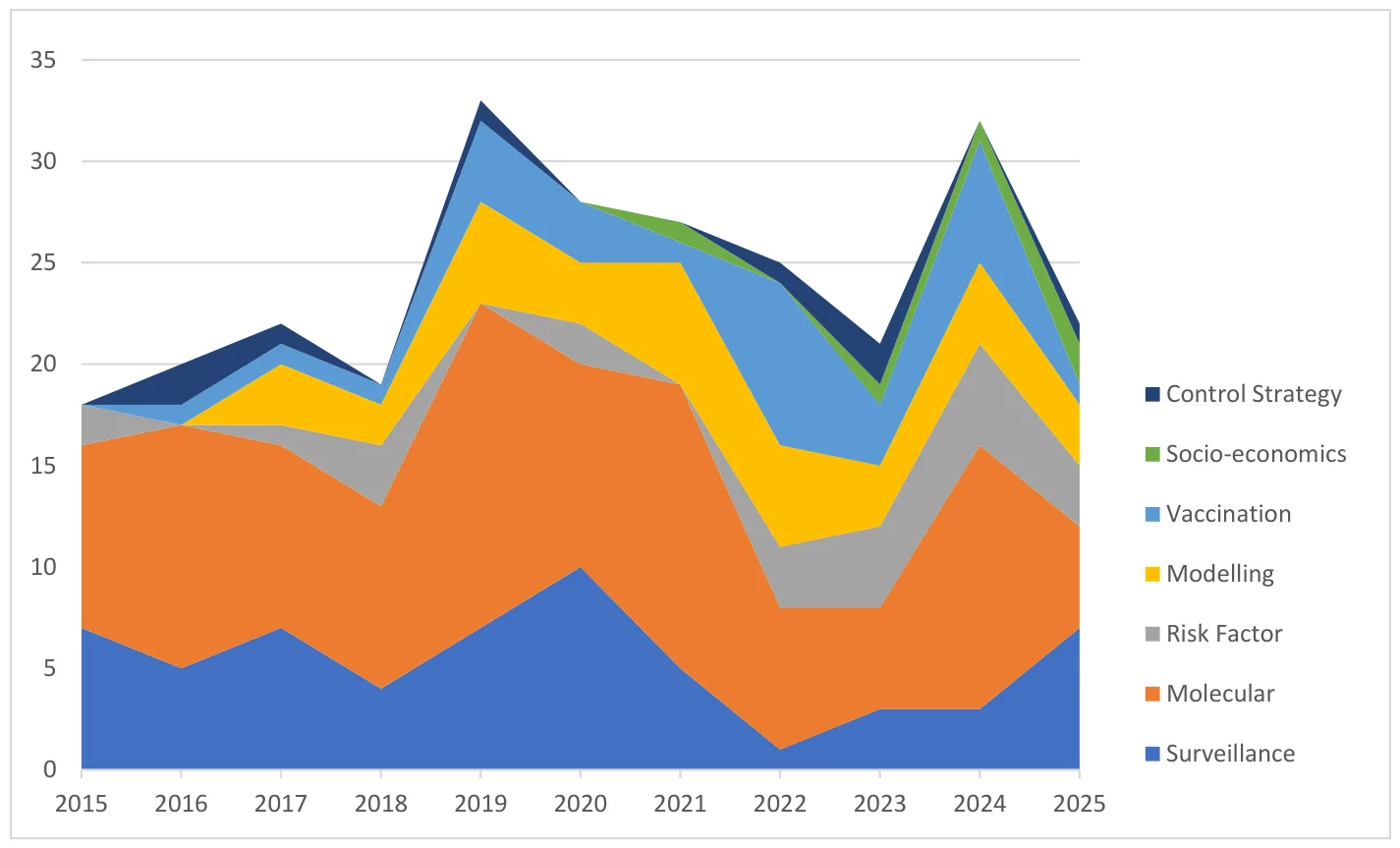
Temporal trends in PPR epidemiology research from 2015 to 2025.

There was a gradual increase in yearly PPR research output over the decade; from 2015 to 2018, publications ranged from 18 to 22 studies per year (6.6–8.1%), rising notably in 2019–2020 with 33 and 32 studies per year (12.1% and 11.7%), respectively, then falling (Figure 3). Molecular epidemiology consistently dominated annual output, with notable peaks in 2016 (*n* = 12), 2019 (*n* = 16), 2021 (*n* = 14), and 2024 (*n* = 13). Surveillance and disease monitoring studies showed moderate but variable activity, peaking in 2020 (*n* = 10) and remaining relatively stable in earlier (2015, 2017, 2019) and later years (2025). However, a sharp decline was observed in 2022 (*n* = 1), followed by modest recovery thereafter.

Analytical and risk-factor studies remained limited throughout the decade, with gradual increases in recent years, particularly in 2023 (*n* = 4) and 2024 (*n* = 5). Modelling and spatial analyses demonstrated intermittent growth beginning in 2017 (*n* = 3), with higher activity observed in 2019 (*n* = 5), 2021 (*n* = 6), and 2022 (*n* = 5). Vaccination epidemiology remained sporadic but showed episodic increases, most notably in 2022 (*n* = 8) and 2024 (*n* = 6).

Socioeconomics studies appeared only from 2021 onward and remained low in number (maximum *n* = 2 in 2025). Control strategy studies were rare throughout the period, with small peaks in 2016 (*n* = 2) and 2023 (*n* = 2).

#### 3.3.2. Geographic Distribution

**Figure 4:**
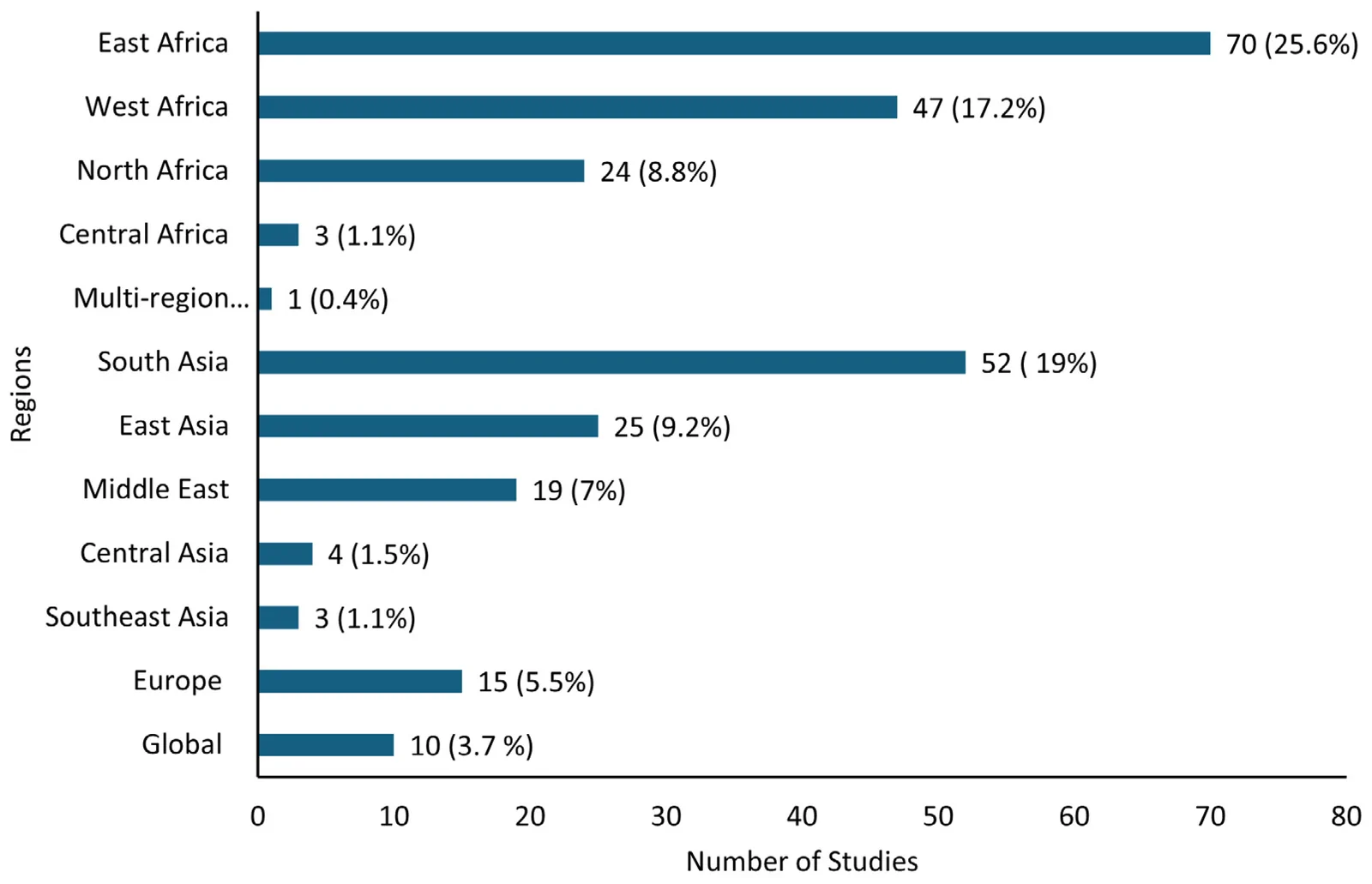
Number of PPR epidemiological studies by region.

As shown in Figure 4, more than half of the included studies were conducted in Africa (*n* = 145; 53.1% of all studies). Within Africa, East Africa accounted for 70 studies (25.6%), followed by West Africa (*n* = 47; 17.2%), North Africa (*n* = 24; 8.8%), Central Africa (*n* = 3; 1.1%), and multi-region African studies (*n* = 1; 0.4%). Asia contributed 103 studies (37.7%), with South Asia representing 52 studies (19.0%), East Asia 25 (9.2%), the Middle East 19 (7.0%), Central Asia 4 (1.5%), and Southeast Asia 3 (1.1%). Europe accounted for 15 studies (5.5%), while globally focused studies comprised 10 publications (3.7%).

Table 3 presents the distribution of studies across major endemic regions by domain. It reveals significant regional clustering and thematic imbalance. Africa accounts for the largest share of studies, with strong representation in East and West Africa across molecular, surveillance, and vaccination domains, while Central Africa is notably underrepresented. In Asia, research activity is concentrated in South Asia and parts of East Asia, particularly in molecular epidemiology and surveillance, whereas Southeast Asia and Central Asia show minimal thematic coverage. Europe and global multi-region analyses contribute only marginally and are primarily focused on molecular studies, modelling, and select control strategy studies.

### 3.4. Progress Across PPR Epidemiological Domains in Relation to GREN Priorities

Molecular epidemiology and surveillance-related research dominated the evidence base and demonstrated substantial methodological advancement and expanding geographic coverage. In contrast, comparatively limited attention has been directed toward vaccination epidemiology, control strategy evaluation, and socioeconomic research despite their central relevance to operational eradication planning. To better interpret how the evolving evidence base aligns with the operational and strategic needs of the PPR GEP, the identified domains were comparatively synthesized in relation to research priorities identified by PPR GREN. Table 4 summarizes comparative progress, remaining gaps, and alignment with GREN priority areas across the major epidemiological domains.

#### 3.4.1. Molecular Epidemiology and Virus Strain Characterization

Molecular epidemiology dominates the literature, accounting for 109 studies (40% of the total). These studies employ advanced phylogenetic and molecular typing approaches to characterize circulating lineages, trace likely sources of incursions, and investigate genetic relationships across regions [17,18,19]. Molecular analyses have been particularly informative in investigating new incursions into previously free countries and have, to some extent, compensated for the relative scarcity of classical field epidemiology studies [20,21]. Rapid advances in sequencing technologies have enabled deeper viral characterization at lower cost and with reduced infrastructure requirements, facilitating broader application in endemic settings [22,23,24,25]. A subset of studies integrates molecular and classical epidemiological approaches to provide additional insights into transmission dynamics, including investigation of wildlife–domestic interfaces and identification of co-infections [26,27,28,29,30].

Despite substantial methodological advances, important gaps remain in systematic virus typing, structured epi-zone definition, and integration of phylogenetic evidence into adaptive risk-based vaccination and surveillance frameworks. Overall, the domain demonstrated advanced methodological maturity but only partial integration into adaptive eradication planning.

#### 3.4.2. Surveillance, Disease Monitoring, and Risk Factor Epidemiology

Surveillance, disease monitoring, and risk factor epidemiology collectively constituted a major component of the PPR evidence base and aligned closely with GREN priorities related to surveillance strengthening, risk-based control, and improved understanding of transmission contexts. This integrated domain accounted for 88 studies (32.2%) of the mapped literature, making it one of the largest research areas after molecular epidemiology. Consistent with broader PPR research trends, serology overwhelmingly dominated the evidence base, with most studies employing cross-sectional seroprevalence or serosurveillance designs to estimate geographic distribution, population exposure, and post-vaccination immunity [31,32]. The literature spanned diverse endemic settings across East, West, and North Africa, South Asia, the Middle East, and parts of Europe, frequently focusing on pastoral and agro-pastoral production systems (see Table 3). Surveillance activities have increasingly extended beyond small ruminants to include several atypical domestic and wildlife host species [33,34,35,36,37]. Additional investigations included abattoir-based surveillance, border screening of imported animals, outbreak confirmation studies, and limited piloting of syndromic and sentinel-based surveillance systems [38,39,40].

Within this broader surveillance landscape, analytical and risk factor studies contributed important evidence on determinants of PPR exposure at host, herd, and production-system levels. Reported risk factors included age, species composition, herd size, mobility, production system, vaccination status, market connectivity, and border proximity, improving understanding of transboundary transmission dynamics and high-risk livestock systems [37,41,42,43,44].

Despite broad geographic coverage and increasing analytical breadth, the domain remained dominated by cross-sectional serological studies, with limited longitudinal surveillance, incidence-based analysis, and causal inference approaches. Standardized sampling frameworks and integration of surveillance outputs into predictive modelling and adaptive control systems also remained limited. Overall, the domain demonstrated intermediate progress in strengthening descriptive surveillance and improving understanding of PPR exposure determinants, but important gaps remain in longitudinal epidemiology, causal inference, and integration of surveillance evidence into adaptive risk-based eradication strategies.

#### 3.4.3. Transmission Dynamics and Risk Modelling

Transmission dynamics and risk modelling accounted for 34 studies (12.5%) and represented one of the most technically advanced and increasingly diversified domains within PPR epidemiology. Studies applied a wide range of approaches, including stochastic and agent-based simulations, ecological niche modelling, machine learning prediction, spatio-temporal analysis, livestock movement network analysis, and import risk assessment frameworks [45,46,47,48,49,50]. These approaches were used to investigate outbreak spread, mobility-driven transmission, vaccination scenarios, elimination feasibility, and high-risk transmission corridors, including integration of climate suitability and livestock movement data [51,52,53].

Despite this analytical breadth, important constraints remain. Many models are calibrated to retrospective outbreak datasets or subnational case reports, with limited high-resolution longitudinal incidence data for validation. Ecological niche and spatial prediction models often rely on presence-only or reported outbreak data of variable completeness, particularly in endemic settings with weak passive surveillance. While hotspot identification and risk mapping are increasingly sophisticated, prospective validation and operational embedding within risk-based surveillance systems are uncommon. Moreover, modelling efforts remain geographically clustered (e.g., China, Central Asia, East Africa), limiting generalizability across diverse production systems.

Overall, transmission dynamics and risk modelling demonstrated substantial methodological progress and increasing relevance to GREN priorities related to risk-based control and adaptive eradication planning. However, limited availability of longitudinal surveillance data, weak institutional integration, and uneven geographic representation continue to constrain operational uptake within endemic-country decision support systems.

#### 3.4.4. Vaccination, Control Strategy, and Socioeconomic Dimensions of PPR Eradication

Vaccination epidemiology, control strategy evaluation, and socioeconomic research collectively represent some of the most operationally relevant yet comparatively underdeveloped domains within the PPR evidence base in relation to GREN research priorities. Although these domains directly address eradication feasibility, implementation effectiveness, and sustainability of control programmes, they remain limited in volume, geographic breadth, and integration into adaptive eradication planning.

Vaccination epidemiology has evolved from descriptive post-vaccination sero-monitoring to integrated analyses of herd immunity dynamics, vaccination scheduling, and socioeconomic factors influencing vaccine uptake. Studies highlighted the importance of demographic turnover, livestock mobility, cold-chain limitations, and uneven campaign implementation in shaping population immunity, while growing socioeconomic evidence emphasized barriers related to service accessibility, gender inequities, willingness to vaccinate, and institutional trust [32,54,55,56,57,58,59]. These findings align closely with GREN priorities on vaccination optimization and implementation-oriented research. However, the evidence base remains dominated by cross-sectional studies, with limited longitudinal evaluation and weak integration of biological, operational, and socioeconomic dimensions into adaptive eradication strategies.

Control strategy evaluation and socioeconomic research remained particularly limited despite their high operational relevance. Existing studies demonstrated important advances in economic impact assessment, benefit–cost analysis, and system dynamics modelling [1,3,60], consistently indicating that PPR control and eradication are capable of generating substantial livelihood benefits. Socioeconomic investigations additionally broadened understanding of farmer perceptions, participatory priority setting, behavioural determinants of vaccine uptake, and gendered barriers within livestock health systems. Nevertheless, most socioeconomic studies remained descriptive and cross-sectional, with limited integration into epidemiological modelling, vaccination evaluation, or operational decision-support systems. Comparative evaluations of alternative control strategies, real-world programme effectiveness, and implementation efficiency remain rare. Overall, these findings suggest that substantial gaps persist in translating operational, economic, and socio-behavioural evidence into integrated, adaptive eradication strategies aligned with GREN priorities.

## 4. Discussion

### 4.1. Overall Imbalance in the PPR Epidemiological Evidence Base

This scoping review demonstrates that the global PPR epidemiological evidence base has expanded substantially over the past decade, reflecting increased international commitment to the PPR GEP and growing research investment across endemic regions. However, the distribution of evidence remains highly uneven across epidemiological domains. Molecular epidemiology and surveillance research dominate the literature, whereas comparatively limited attention has been directed toward vaccination epidemiology, operational control strategy evaluation, and socioeconomic and implementation-focused epidemiology.

This imbalance is important because the epidemiological requirements for early-stage disease characterization differ substantially from those required for the final stages of eradication. While molecular characterization and serological mapping remain essential, molecular characterization is also a critical component of laboratory diagnosis, outbreak confirmation, and surveillance systems that underpin effective PPR control and eradication efforts. The substantial body of research in this area has therefore made an important contribution to the eradication agenda by enabling virus detection, lineage tracking, and improved understanding of disease spread across endemic regions. Eradication increasingly depends on operationally integrated evidence capable of informing adaptive vaccination, surveillance optimization, resource prioritization, and implementation effectiveness under diverse production systems [9,61]. The current evidence landscape, therefore, suggests that scientific activity has expanded more rapidly in laboratory and descriptive domains than in implementation-oriented epidemiology, highlighting the need to complement these indispensable laboratory advances with stronger operational, modelling, socioeconomic, and implementation research that directly supports eradication delivery.

### 4.2. Dominance of Molecular Epidemiology and Descriptive Surveillance

Molecular epidemiology represented the largest proportion of studies identified in this review and demonstrates the most advanced methodological development. The widespread application of phylogenetic analysis and expanding access to sequencing technologies have substantially improved the understanding of lineage distribution [17,18], transboundary circulation, and outbreak tracing across endemic and newly affected regions [62,63]. In several settings, molecular analyses have compensated for limitations in field epidemiological data by providing indirect evidence of transmission connectivity and geographic spread [18,64,65].

Similarly, surveillance and disease monitoring studies have generated extensive serological evidence across Africa, Asia, and the Middle East. These investigations have improved the understanding of population exposure, geographic distribution, and the role of atypical hosts and wildlife species [31,32,66]. While these studies provide extensive descriptive evidence, they primarily measure cumulative exposure instead of incidence, limiting their ability to analyse ongoing transmission dynamics. Inconsistent age adjustment, varying sampling strategies, and a lack of longitudinal seroconversion studies further hinder comparability. Surveillance is often project-based and lacks integration into coordinated national systems. The absence of DIVA-compatible vaccines complicates interpreting seropositivity in vaccinated populations. Ultimately, while surveillance offers valuable insights, its effectiveness in informing real-time transmission assessment and adaptive eradication planning is restricted.

### 4.3. Expansion of Transmission Dynamics and Risk Modelling

Transmission and risk modelling for PPR has expanded considerably over the past decade. The field has evolved substantially beyond classical deterministic compartmental modelling toward increasingly diverse analytical approaches, including stochastic simulation, spatial and ecological suitability modelling, Bayesian spatio-temporal modelling, machine learning, and livestock movement network analysis [45,49,50]. These approaches have improved understanding of transmission heterogeneity, demographic turnover, cross-border spread, environmental suitability, and vaccination threshold dynamics across multiple endemic settings. However, challenges remain, such as reliance on low-resolution data and a lack of prospective validation. Although risk maps are becoming more sophisticated, modelling efforts are limited to a small, fragmented community, affecting continuity and broader application in veterinary decision-making.

Risk factor studies have enhanced the understanding of determinants of PPR exposure [37,42,44]. However, the field is still largely dominated by cross-sectional, serology-based studies using associative statistics. Longitudinal cohort studies, which offer better insights into disease patterns than cross-sectional studies, are rare. Spatial analysis is inconsistently utilized, and connections between risk factors and predictive modelling are limited. Geographic coverage is uneven, concentrated in a few countries, with few harmonized, multi-site designs. While many cross-sectional studies identify associations with outbreaks, they mainly reinforce poor biosecurity and practices that increase pathogen exposure, leading to higher disease rates.

The findings indicate that the main challenge in this field is no longer the development of methods but rather the integration of institutions [67,68]. Enhancing the connections between modelling platforms, standardized surveillance systems, and national eradication programs could significantly improve the practical application of transmission modelling within adaptive PPR control frameworks.

### 4.4. Persistent Operational and Socioeconomic Gaps in Vaccination and Control Strategy Evaluation

One of the most important findings of this review is the relative underdevelopment of vaccination epidemiology and control strategy evaluation despite their central relevance to eradication feasibility. Although modelling studies and post-vaccination sero-monitoring have improved understanding of herd immunity dynamics, demographic turnover, and vaccination coverage challenges [32,56], relatively few studies evaluate the operational effectiveness of alternative vaccination delivery strategies under field conditions.

Evidence from transmission models consistently demonstrates that livestock turnover rapidly reduces effective population immunity in endemic systems, particularly in pastoral and agro-pastoral production settings [49]. These findings reinforce the importance of sustained vaccination efforts, strategic campaign timing, and geographically targeted interventions. However, empirical evaluation of vaccination delivery systems, campaign efficiency, cold-chain performance, and implementation barriers remains limited.

Similarly, control strategy evaluation remains one of the least represented areas identified in this review. Existing studies largely focus on economic justification and broad eradication feasibility rather than comparative assessment of operational strategies [69,70]. Comparative empirical evaluation of delivery strategies, such as mass versus targeted approaches, optimal campaign frequency, or integration with surveillance, is scarce. Much of the evidence remains theoretical or model-based rather than grounded in field implementation. As eradication efforts progress, stronger implementation-oriented evidence will likely become increasingly important for optimizing resource allocation, targeting high-risk populations, and evaluating alternative delivery approaches across heterogeneous livestock systems.

Socioeconomic research remains limited in volume but demonstrates greater conceptual and operational relevance than previously recognized. Existing studies extend beyond simple perception surveys to examine farmer knowledge and attitudes, gendered dimensions of disease management, socioeconomic drivers of disease emergence and spread, willingness to vaccinate (WTV), willingness to pay (WTP), and participatory approaches to priority setting [71,72,73,74,75]. Collectively, these studies highlight that vaccination uptake and surveillance participation are shaped not only by vaccine availability but also by institutional trust, service accessibility, livelihood constraints, market incentives, and social inequalities. However, the evidence base remains methodologically fragmented and predominantly descriptive, with limited use of longitudinal, mixed-methods, or implementation-oriented designs. As eradication efforts progress toward the final stages of control, stronger integration of socioeconomic evidence into epidemiological planning and programme evaluation will become increasingly important for improving campaign effectiveness, targeting underserved populations, and strengthening the sustainability of PPR eradication strategies.

### 4.5. Implications for Adaptive Eradication Planning and GREN Priorities

The comparative synthesis across domains highlights several research priorities that remain incompletely addressed within the current evidence base. These include systematic definition of epi-zones, longitudinal measurement of transmission dynamics, operational evaluation of vaccination strategies, integration of wildlife and atypical host surveillance, and incorporation of socioeconomic evidence into programme design.

Importantly, many of the remaining challenges identified in this review are not solely technical but institutional and operational. The limited embedding of modelling outputs, surveillance evaluation, and implementation research within national veterinary systems constrains the translation of epidemiological evidence into adaptive eradication strategies. Strengthening this research-to-policy interface may therefore be as important as generating additional descriptive data. Evidence from successful animal disease control programmes, particularly the global eradication of rinderpest, demonstrates that sustained collaboration among researchers, veterinary services, policymakers, and international partners facilitates the translation of scientific evidence into surveillance, vaccination, and disease control policies. Strengthening similar research–policy partnerships through the PPR-GREN and national veterinary systems could enhance evidence-informed decision-making and improve implementation of adaptive eradication strategies.

The findings of this review also have important implications for the PPR-GEP target of global eradication by 2030. While the 2030 target has been instrumental in mobilizing international commitment, investment, and coordinated action, the persistent research gaps identified in implementation epidemiology, disease modelling, operational research, and socioeconomic evidence suggest that achieving global eradication within the original timeline remains challenging in many endemic settings. Addressing these gaps, together with sustained political commitment, adequate investment, and adaptive programme implementation, will be critical to maintaining progress toward the ultimate goal of global PPR eradication, even where continued efforts beyond the current programme timeline may be required.

Future progress toward eradication will likely require a transition from predominantly descriptive epidemiology toward more integrated implementation-oriented research frameworks capable of supporting real-time decision-making under heterogeneous endemic conditions.

### 4.6. Limitations of the Study

This review provides a comprehensive synthesis of epidemiological research relating to PPR published between 2015 and 2025. It is important to acknowledge some limitations of this review. It primarily relied on published peer-reviewed literature, which means that significant operational experiences, program reports, and grey literature produced by national veterinary services and partners involved in the FAO-WOAH PPR Global Eradication Programme may not have been fully represented.

## 5. Conclusions

The accumulated body of PPR research over the past decade demonstrates that the global PPR epidemiological evidence base has expanded substantially and demonstrates important methodological advances, particularly in molecular epidemiology and surveillance. However, critical gaps remain in the domains most directly relevant to eradication implementation. To effectively address these gaps, there needs to be greater integration across epidemiological, operational, and socioeconomic disciplines. This will support the development of more responsive and evidence-informed eradication strategies that can be adapted to various endemic settings. Additionally, there is a need to better integrate the research–policy cycle, where research questions are driven by policy needs, and research findings directly inform policy decisions. Achieving this will require strong collaboration between the research and government sectors at both national and sub-national levels. The successful eradication of rinderpest demonstrated the value of a strong research–policy interface, where evidence-based decision-making and close coordination among researchers, veterinary authorities, and policymakers were instrumental in achieving eradication goals.

## Figures and Tables

**Table 1:** Epidemiological domain classification framework.

Domain	Operational Definition	Typical Study Designs/Analytical Approaches
Analytical and Risk Factor	Studies assessing determinants, risk factors, or drivers of PPR occurrence using inferential epidemiological analysis.	Cross-sectional, case–control, cohort studies; multivariable regression; odds ratios; risk ratios; association modelling.
Surveillance and Disease Monitoring	Studies reporting disease monitoring systems, outbreak investigations, and descriptive epidemiology of PPR occurrence.	Passive or active surveillance; outbreak reports; prevalence/incidence estimation; temporal trend analysis.
Transmission dynamics and risk modelling	Studies applying statistical, mathematical, or spatial methods to analyse transmission dynamics, predict spread, or map risk.	Transmission models (e.g., SIR-type); spatial risk mapping; geostatistics; predictive modelling; scenario simulations.
Molecular and Virus Strain Characterization	Genetic and phylogenetic investigations examining viral characterization, lineage distribution, and molecular transmission dynamics.	Genome sequencing; phylogenetic analysis; molecular clustering; evolutionary analysis.
Vaccination	Studies evaluating vaccine performance, immunological response at the population level, and vaccination impact.	Vaccine effectiveness studies; coverage assessments; post-vaccination sero-monitoring; impact analysis.
Control Strategy Evaluation	Assessment of intervention programs, eradication strategies, policy implementation, and economic evaluation of control measures.	Program evaluation; cost-effectiveness analysis; policy analysis; impact assessment frameworks.
Socioeconomic	Studies examining socioeconomic determinants, behavioural factors, livestock mobility, and value chain dynamics influencing transmission.	KAP surveys; participatory epidemiology; value chain analysis; mobility network studies.
Evidence Synthesis	Structured synthesis of epidemiological evidence addressing PPR distribution, determinants, or control.	Systematic reviews; meta-analyses; structured evidence mapping.

**Table 2:** Summary of the characteristics of the included studies.

Characteristic	Category	Number of Studies	Percent of Total Studies (%)
Publication Period	2015–2017	60	20.0
	2018–2020	85	31.1
	2021–2023	73	26.7
	2024–2025	55	20.2
Geographic Region	Africa	145	53.1
	Asia	103	37.7
	Europe	15	5.5
	Global	10	3.7
Species Studied	Domestic	258	94.5
	Wildlife	15	5.5
Primary Epidemiology Domain	Molecular Epidemiology	109	40.0
	Surveillance and Disease Monitoring	59	21.6
	Analytical and Risk Factor	23	8.4
	Transmission Dynamics and Risk Modelling	34	12.5
	Vaccination Epidemiology	29	10.6
	Control Strategy Evaluation	8	2.9
	Socioeconomics Research	5	1.8
	Evidence Synthesis	6	2.2
Diagnosis Methods	PCR/Sequencing	94	34.4
	Serology	83	30.4
	Mixed Biological Methods	17	6.2
	Secondary Data/Database	48	17.6
	Social Survey	28	10.3
	Clinical/Field	3	1.1

**Table 3:** Geographic distribution of studies by epidemiological domain (2015–2025).

	Regions	Regions	Analytical and Risk Factor	Surveillance and Disease Monitoring	Transmission Dynamics and Risk Modelling	Molecular and Virus Strain Characterization	Socioeconomic	Vaccination
Africa	North Africa	3	11	1	7	1	1	0
	West Africa	1	10	3	23	0	7	3
	Central Africa	1	0	0	2	0	0	0
	East Africa	15	14	10	14	2	14	1
	Multi-region	0	0	0	1	0	0	0
Asia	Middle East	0	6	1	12	0	0	0
	Central Asia	0	1	2	1	0	0	0
	East Asia	0	2	6	17	0	0	0
	South Asia	3	14	4	23	1	6	1
	Southeast Asia	0	3	0	0	0	0	0
Europe	Europe	0	2	4	8	1	0	0
Global	Global	0	2	3	1	0	1	3

Note: Cell shading represents the relative magnitude of the values within the table; darker blue indicates higher numbers of studies, whereas lighter blue indicates lower numbers.

**Table 4:** Comparative progress across PPR epidemiological domains (2015–2025) in relation to GREN priorities (2015–2025).

Domain	Number of Studies (%)	Major Advances	Key Remaining Gaps	GREN-Relevant Priority Gaps	Relative Progress
Molecular Epidemiology	109 (40.0%)	Expanded phylogenetic characterization; lineage tracking; improved outbreak tracing; wider sequencing capacity	Limited integration with livestock movement and longitudinal epidemiology; opportunistic sampling	Weak epi-zone definition; limited wildlife transmission integration	Advanced
Surveillance and Disease Monitoring	65 (23.8%)	Broad serological surveillance across endemic regions; increasing atypical host investigations	Predominantly cross-sectional studies; limited incidence estimation and surveillance evaluation	Weak integration into adaptive risk-based surveillance and eradication planning	Intermediate
Transmission Dynamics and Risk Modelling	34 (12.5%)	Expansion of spatial modelling, network analysis, ecological suitability mapping, and simulation models	Limited longitudinal validation; geographically clustered applications; weak operational uptake	Limited integration into vaccination targeting and surveillance optimization	Intermediate
Analytical & Risk Factor Epidemiology	23 (8.4%)	Improved identification of host-, herd-, and system-level risk factors	Predominantly associative cross-sectional studies; limited causal inference	Limited translation into targeted surveillance and vaccination strategies	Emerging
Vaccination (vaccine) epidemiology	29 (10.6%)	Improved understanding of herd immunity turnover, demographic replacement, and vaccine uptake barriers	Limited longitudinal vaccine effectiveness studies and operational delivery evaluation	Weak evidence for optimizing vaccination schedules, delivery systems, and immunity monitoring	Emerging
Control Strategy Evaluation	8 (2.9%)	Economic and systems-based analyses support eradication feasibility	Very limited empirical evaluation of operational strategies and implementation effectiveness	Minimal comparative assessment of vaccination strategies and campaign efficiency	Emerging
Socioeconomic Research	5 (1.8%)	Improved understanding of farmer perceptions, WTV/WTP, gender dynamics, and institutional barriers	Small and methodologically fragmented evidence base	Limited integration into surveillance, vaccination delivery, and policy design	Emerging

## Data Availability

The original contributions presented in this study are included in the article and Supplementary Materials. Further inquiries can be directed to the corresponding author.

## Supplementary Materials

The following supporting information can be downloaded at https://www.mdpi.com/article/10.3390/v18080857/s1, Table S1: PRISMA-ScR checklist; Table S2: Characteristics of all studies included in the scoping review (*n* = 273).

## References

[1] Jones B.A., Rich K.M., Mariner J.C., Anderson J., Jeggo M., Thevasagayam S., Cai Y., Peters A.R., Roeder P. (2016). The Economic Impact of Eradicating Peste des Petits Ruminants: A Benefit-Cost Analysis. PLoS ONE.

[2] FAO, OIE (2016). Peste des Petits Ruminants Global Eradication Programme Contributing to Food Security, Poverty Alleviation, and Resilience; Five Years (2017–2021).

[3] Meyer A., Ndiaye B., Larkins A., Chaters G., Gilbert W., Huntington B., Huntington B., Ilboudo G., Dione M., Jemberu W.T. (2025). Economic assessment of animal disease burden in Senegalese small ruminants. Prev. Vet. Med..

[4] Jemberu W.T., Knight-Jones T.J.D., Gebru A., Mekonnen S.A., Yirga A., Sibhatu D., Rushton J. (2022). Economic impact of a peste des petits ruminants outbreak and vaccination cost in northwest Ethiopia. Transbound. Emerg. Dis..

[5] OIE, FAO (2015). Global Strategy for the Control and Eradication of PPR.

[6] Peters M.D.J., Marnie C., Tricco A.C., Pollock D., Munn Z., Alexander L., McInerney P., Godfrey C.M., Khalil H. (2021). Updated methodological guidance for the conduct of scoping reviews. JBI Evid. Implement..

[7] Tricco A.C., Lillie E., Zarin W., O’Brien K.K., Colquhoun H., Levac D., Moher D., Peters M.D.J., Horsley T., Weeks L. (2018). PRISMA extension for scoping reviews (PRISMA-ScR): Checklist and explanation. Ann. Intern. Med..

[8] FAO, WOAH (2022). Peste des Petits Ruminants Global Eradication Programme II & III: Overview of the Plan of Action.

[9] FAO, OIE (2021). Proceedings of the PPR Global Research and Expertise Network (PPR-GREN): 3rd Meeting, Report of Virtual Meeting, 9–12 November 2020.

[10] FAO, OIE (2018). Proceedings of the PPR Global Research and Expertise Network (PPR-GREN) Inaugural Meeting, Vienna, Austria, 17–19 April 2018.

[11] FAO, OIE (2021). Proceedings of the 4th Meeting of the Peste des Petits Ruminants Global Research and Expertise Network (PPR-GREN IV), Virtual Zoom Meeting, 6–8 December 2021.

[12] WOAH, FAO (2022). Proceedings of the PPR Global Research And Expertise Network (PPR-GREN) Fifth Meeting Montpelier, Montpelier, France, 7–9 December 2022.

[13] FAO, WOAH (2023). Proceedings of the Report of the Annual meeting of the Peste des Petits Ruminants Global Research and Expertise Network, Bangalore, India, 28–30 November 2023.

[14] WOAH, FAO (2025). Proceedings of the 7th Annual Meeting of the Peste des Petits Ruminants Global Research and Expertise Network, Abu Dhabi, UAE, 20–22 January 2025.

[15] FAO, OIE (2019). Proceedings of the PPR Global Research and Expertise Network (PPR-GREN): Second Meeting, Nairobi, Kenya, 13–15 November 2019.

[16] Page M.J., McKenzie J.E., Bossuyt P.M., Boutron I., Hoffmann T.C., Mulrow C.D., Shamseer L., Tetzlaff J.M., Akl E.A., Brennan S.E. (2021). The PRISMA 2020 statement: An updated guideline for reporting systematic reviews. PLoS Med..

[17] Alemu B., Gari G., Libeau G., Kwiatek O., Kidane M., Belayneh R., Siraw B., Wieland B., Asfaw W., Abdi R.D. (2019). Molecular detection and phylogenetic analysis of Peste des petits ruminants virus circulating in small ruminants in eastern Amhara region, Ethiopia. BMC Vet. Res..

[18] Rahman M.M., Islam M.S., Sabuj A.A.M., Hossain M.G., Islam M.A., Alam J., Ershaduzzaman M., Saha S. (2023). Molecular Epidemiology and Phylogenetic Analysis of Peste des Petits Ruminants Virus Circulating in Sheep in Bangladesh. Transbound. Emerg. Dis..

[19] Saeed F.A., Gumaa M., AAbdelaziz S., Enan K.A., Ahmed S.K., Hussien M.O. (2021). Epidemiology and molecular characterization of re-emerged virulent strains of Peste des Petits Ruminants virus among sheep in Kassala State, Eastern Sudan. Ir. Vet. J..

[20] Guendouz S., Kwiatek O., Kirtzalidou A., Katsifa A., Gianniou M., Ancuceanu C., Ghiță M., Mortasivu C.L., Zdravkova A., Kostov I. (2025). Genomic analysis of peste des petits ruminants virus in Europe: Common origin for emergence in Greece, Romania, and Bulgaria. Infect. Genet. Evol..

[21] Clarke B., Mahapatra M., Friedgut O., Bumbarov V., Parida S. (2017). Persistence of Lineage IV Peste-des-petits ruminants virus within Israel since 1993: An evolutionary perspective. PLoS ONE.

[22] Muniraju M., Mahapatra M., Ayelet G., Babu A., Olivier G., Munir M., Libeau G., Batten C., Banyard A.C., Parida S. (2016). Emergence of Lineage IV Peste des Petits Ruminants Virus in Ethiopia: Complete Genome Sequence of an Ethiopian Isolate 2010. Transbound. Emerg. Dis..

[23] Chukwudi I.C., Mira F., Ogbu K.I., Malesa R.P., Purpari G., Luka P.D., Ugochukwu E.I., Heath L.E., Guercio A., Chah K.F. (2021). Peste des petits ruminants in Nigeria: Identification and variations of lineage II and lineage IV in sheep and goats. Vet. Ital..

[24] Kinimi E., Mahapatra M., Kgotlele T., Makange M.R., Tennakoon C., Njeumi F., Odongo S., Muyldermans S., Kock R., Parida S. (2021). Complete genome sequencing of field isolates of peste des petits ruminants virus from tanzania revealed a high nucleotide identity with lineage III ppr viruses. Animals.

[25] Chukwudi I.C., Ogbu K.I., Luka P.D., Malesa R.P., Heath L.E., Ugochukwu E.I., Chah K.F. (2020). Comparison of colorimetric loop-mediated isothermal amplification kit and reverse transcription-polymerase chain reaction in the diagnosis of peste des petits ruminants in sheep and goats in Southeast Nigeria. Vet. World.

[26] Sultana S., Pervin M., Sultana N., Siddique M.P., Islam M.R., Khan M.A.H.N.A. (2022). Identification of peste des petits ruminants virus along with co-infecting diseases of goats in Bangladesh. J. Adv. Vet. Anim. Res..

[27] Wasee Ullah R., Bin Zahur A., Latif A., Iqbal Dasti J., Irshad H., Afzal M., Rasheed T., Rashid Malik A., Qureshi Z.U.A. (2016). Detection of Peste des Petits Ruminants Viral RNA in Fecal Samples of Goats after an Outbreak in Punjab Province of Pakistan: A Longitudinal Study. Biomed. Res. Int..

[28] Benfield C.T.O., Hill S., Shatar M., Shiilegdamba E., Damdinjav B., Fine A., Willett B., Kock R., Bataille A. (2021). Molecular epidemiology of peste des petits ruminants virus emergence in critically endangered Mongolian saiga antelope and other wild ungulates. Virus Evol..

[29] Ishag H.Z.A., Terab A.M.A., Eltahir Y.M., El Tigani-Asil E.T.A., Khalil N.A.H., Gasim E.F.M., Yuosf M.F., Al Yammahi S.M.S., Al Mansoori A.M.A., Al Muhairi S.S.M. (2023). A Clinical, Pathological, Epidemiological and Molecular Investigation of Recent Outbreaks of Peste des Petits Ruminants Virus in Domestic and Wild Small Ruminants in the Abu Dhabi Emirate, United Arab Emirates. Vet. Sci..

[30] Moudgil P., Kumar R., Jangir B.L., Gupta R., Vaishali Jindal N. (2022). Epidemiology, risk factors and molecular characterization of small ruminant morbillivirus in Haryana, India. Res. Vet. Sci..

[31] Yirga A., Jemberu W.T., Lyons N., Gebru A., Akililu F., Rushton J. (2020). Post-vaccination herd immunity against peste des petits ruminants and inter-vaccination population turnover in small ruminant flocks in northwest Ethiopia. Prev. Vet. Med..

[32] Mandefro M., Ibrahim S.M., Sibhatu D., Kassa N., Emiyu K., Debebe K., Dessalegn B., Birhan M., Bitew M. (2024). Vaccine sero-monitoring and sero-surveillance of Peste des petits ruminants in small ruminants in West Gojjam zone, Amhara region, Ethiopia. Front. Vet. Sci..

[33] Mahapatra M., Sayalel K., Muniraju M., Eblate E., Fyumagwa R., Shilinde S., Maulidmdaki M., Keyyu J., Parida S., Kock R. (2015). Spillover of Peste des Petits Ruminants Virus. Emerg. Infect. Dis..

[34] Sendow I., Hoerudin H., Hartawan R., Fairusya N., Ratnawati A., Wardhana A.H., Sawitri D.H., Nuradji H., Dharmayanti N.L.P.I., Saepulloh M. (2024). Seroprevalence of peste des petits ruminants disease in Indonesian buffaloes may be an emerging threat to small ruminants. Vet. World.

[35] Jones B.A., Mahapatra M., Mdetele D., Keyyu J., Gakuya F., Eblate E., Lekolool I., Limo C., Ndiwa J.N., Hongo P. (2021). Peste des petits ruminants virus infection at the wildlife–livestock interface in the greater serengeti ecosystem, 2015–2019. Viruses.

[36] Chowdhury E.H., Kundu P., Begum J.A., Chowdhury T., Rahman M., Khatun A., Saha S.S., Nooruzzaman M., Parvin R., Islam M.R. (2022). Peste des petits ruminants virus antibodies in domestic large ruminants in Bangladesh. J. Infect. Dev. Ctries..

[37] Nkamwesiga J., Lumu P., Nalumenya D.P., Korennoy F., Roesel K., Wieland B., Perez A., Kiara H., Muhanguzi D. (2023). Seroprevalence and risk factors of Peste des petits ruminants in different production systems in Uganda. Prev. Vet. Med..

[38] Cosseddu G.M., Doumbia B., Scacchia M., Pinoni C., Di Provvido A., Polci A., Isselmou K., Di Gennaro A., Spedicato M., Carmine I. (2021). Sero-surveillance of emerging viral diseases in camels and cattle in Nouakchott, Mauritania: An abattoir study. Trop. Anim. Health Prod..

[39] Lysholm S., Lindahl J.F., Munyeme M., Misinzo G., Mathew C., Alvåsen K., Dautu G., Linde S., Mitternacht L., Olovsson E. (2022). Crossing the Line: Seroprevalence and Risk Factors for Transboundary Animal Diseases Along the Tanzania-Zambia Border. Front. Vet. Sci..

[40] Balogun F.A., Fasanmi O.G., Oladipo T.A., Popoola M.A., Olona J.F., Adeoye Y.D. (2017). Field evaluation and confirmation of acute peste des petits ruminant outbreak in a flock of West African dwarf goats in Ibadan, Nigeria. Int. J. Vet. Sci. Med..

[41] Dayhum A., Sharif M., Eldaghayes I., Kammon A., Calistri P., Danzetta M.L., Di Sabatino D., Petrini A., Ferrari G., Grazioli S. (2018). Sero-prevalence and epidemiology of peste des petits ruminants in Libya. Transbound. Emerg. Dis..

[42] Moumin G., Moussa C., Teshale S., Gezahegne M. (2018). Seroprevalence and risk factors for peste des petits ruminants in sheep and goats in Djibouti. Rev. Sci. Tech..

[43] Gelana M., Gebremedhin E.Z., Gizaw D. (2020). Seroepidemiology of Peste des Petits ruminants in sheep and goats in the selected district of Horu Guduru Zone, Western Ethiopia. Res. Vet. Sci..

[44] Abesha H., Teshome Y., Alemu Y.F., Dejene H., Tarekegn Z.S., Assefa A. (2023). Seroepidemiology of peste des petits ruminants virus in small ruminants in selected districts in Northwest Ethiopia. Vet. Med. Sci..

[45] Ashango A.A., Waktole H., Leta S., Negussie H. (2025). Mapping Small Ruminant Trade Networks in Ethiopia’s Somali Region and Borena Zone: Implications for the Spread of Peste des Petits Ruminants Virus. Transbound. Emerg. Dis..

[46] Bouchemla F., Agoltsov V.A., Popova O.M., Padilo L.P. (2018). Assessment of the peste des petits ruminants world epizootic situation and estimate its spreading to Russia. Vet. World.

[47] Niu B., Liang R., Zhou G., Zhang Q., Su Q., Qu X., Chen Q. (2021). Prediction for Global Peste des Petits Ruminants Outbreaks Based on a Combination of Random Forest Algorithms and Meteorological Data. Front. Vet. Sci..

[48] Ruget A.S., Tran A., Waret-Szkuta A., Moutroifi Y.O., Charafouddine O., Cardinale E., Cêtre-Sossah C., Chevalier V. (2019). Spatial Multicriteria Evaluation for Mapping the Risk of Occurrence of Peste des Petits Ruminants in Eastern Africa and the Union of the Comoros. Front. Vet. Sci..

[49] Savagar B., Jones B.A., Arnold M., Walker M., Fournié G. (2023). Modelling flock heterogeneity in the transmission of peste des petits ruminants virus and its impact on the effectiveness of vaccination for eradication. Epidemics.

[50] Yessenbayev K., Mukhanbetkaliyev Y., Yessembekova G., Kadyrov A., Sultanov A., Bainiyazov A., Bakishev T., Nkamwesiga J., Korennoy F., Abdrakhmanov S. (2023). Simulating the Spread of Peste des Petits Ruminants in Kazakhstan Using the North American Animal Disease Spread Model. Transbound. Emerg. Dis..

[51] Assefa A., Tibebu A., Bihon A., Yimana M. (2021). Global ecological niche modelling of current and future distribution of peste des petits ruminants virus (PPRv) with an ensemble modelling algorithm. Transbound. Emerg. Dis..

[52] Nkamwesiga J., Rascón-García K., Lumu P., Kiara H., Perez A., Muhanguzi D., Roesel K. (2025). Network Analysis of Small Ruminant Movements in Uganda: Implications for Control of Transboundary Animal Diseases. Transbound. Emerg. Dis..

[53] Tounkara K., Kwiatek O., Niang M., Abou Kounta Sidibe C., Sery A., Dakouo M., Salami H., Lo M.M., Ba A., Diop M. (2019). Genetic Evidence for Transboundary Circulation of Peste Des Petits Ruminants Across West Africa. Front. Vet. Sci..

[54] Acosta D., Ludgate N., McKune S.L., Russo S. (2022). Who Has Access to Livestock Vaccines? Using the Social-Ecological Model and Intersectionality Frameworks to Identify the Social Barriers to Peste des Petits Ruminants Vaccines in Karamoja, Uganda. Front. Vet. Sci..

[55] Balamurugan V., Varghese B., SowjanyaKumari S., Kumar K.V., Muthuchelvan D., Govindaraj G., Suresh K.P., Hemadri D., Roy P., Shome B.R. (2022). Assessment of post-vaccination immune response to peste des petits ruminants virus in small ruminants in the central and western regions of India. VirusDisease.

[56] Hammami P., Lancelot R., Lesnoff M. (2016). Modelling the dynamics of post-vaccination immunity rate in a population of Sahelian sheep after a vaccination campaign against peste des petits ruminants virus. PLoS ONE.

[57] Ilboudo G.S., Wanyoike F., Bahta S., Sy S., Djigo C.A.T., Sall P.A., Lô M.M., Dione M. (2024). Willingness to vaccinate and willingness to pay for vaccination against peste des petits ruminants in northern Senegal. Front. Vet. Sci..

[58] Walle Y., Mugisha J.Y.T., Melese D., Tessema H. (2024). Modeling the Peste des Petits Ruminants (PPR) disease transmission dynamics with impacts of vaccination and restocking in small ruminant population in Amhara region, Ethiopia. Heliyon.

[59] Williams S., Endacott I., Ekiri A.B., Kichuki M., Dineva M., Galipo E., Alexeenko V., Alafiatayo R., Mijten E., Varga G. (2022). Barriers to vaccine use in small ruminants and poultry in Tanzania. Onderstepoort J. Vet. Res..

[60] Aboah J., Apolloni A., Duboz R., Wieland B., Kotchofa P., Okoth E., Dione M. (2023). Ex-ante impact of pest des petits ruminant control on micro and macro socioeconomic indicators in Senegal: A system dynamics modelling approach. PLoS ONE.

[61] Njeumi F., Bailey D., Soula J.J., Diop B., Tekola B.G. (2020). Eradicating the Scourge of Peste Des Petits Ruminants from the World. Viruses.

[62] Biguezoton A.S., Ilboudo G.S., Wieland B., Sawadogo R.W.Y., Dah F.F., Sidibe C.A.K., Zoungrana A., Okoth E., Dione M. (2024). Molecular Epidemiology of Peste Des Petits Ruminants Virus in West Africa: Is Lineage IV Replacing Lineage II in Burkina Faso?. Viruses.

[63] Niyokwishimira A., de D Baziki J., Dundon W.G., Nwankpa N., Njoroge C., Boussini H., Wamwayi H., Jaw B., Cattoli G., Nkundwanayo C. (2019). Detection and molecular characterization of Peste des Petits Ruminants virus from outbreaks in Burundi, December 2017–January 2018. Transbound. Emerg. Dis..

[64] Iacobescu-Marițescu R., Morar A., Herman V., Tîrziu E., Dégi J., Imre K. (2025). Field-Based Characterization of Peste des Petits Ruminants in Sheep in Romania: Clinical, Pathological, and Diagnostic Perspectives. Vet. Sci..

[65] Rume V.N., Dundon W.G., Belay G., Baziki Jde D., Diakite A., Paul A., Tessema Y.D., Nwankpa N., Gizaw D., Cattoli G. (2019). Molecular epidemiological update of Peste des Petits Ruminants virus (PPRV) in Ethiopia. Vet. Microbiol..

[66] Ahaduzzaman M. (2020). Peste des petits ruminants (PPR) in Africa and Asia: A systematic review and meta-analysis of the prevalence in sheep and goats between 1969 and 2018. Vet. Med. Sci..

[67] Nkamwesiga J., Korennoy F., Lumu P., Nsamba P., Mwiine F.N., Roesel K., Wieland B., Perez A., Kiara H., Muhanguzi D. (2022). Spatio-temporal cluster analysis and transmission drivers for Peste des Petits Ruminants in Uganda. Transbound. Emerg. Dis..

[68] Dione M.M., Traoré I., Kassambara H., Sow A.N., Touré C.O., Sidibé C.A.K., Séry A., Yena A.S., Wieland B., Dakouo M. (2019). Integrated Approach to Facilitate Stakeholder Participation in the Control of Endemic Diseases of Livestock: The Case of Peste Des Petits Ruminants in Mali. Front. Vet. Sci..

[69] Ilboudo G.S., Kane P.A., Kotchofa P., Okoth E., Maiga A., Dione M. (2022). Peste des Petits Ruminants (PPR) Vaccination Cost Estimates in Burkina Faso. Animals.

[70] Lyons N.A., Jemberu W.T., Chaka H., Salt J.S., Rushton J. (2019). Field-derived estimates of costs for Peste des Petits Ruminants vaccination in Ethiopia. Prev. Vet. Med..

[71] Kodo Z.M., Rayyanu U.A., Olabode M.P., Ifende V.I., Weka R.P., Gukut M.Y., Delabouglise A., Shittu I., Atuman Y.J., Bakam J.D. (2025). When farmers’ knowledge matters: Improving epidemiological understanding of Peste des petits ruminants in northern Nigeria. Prev. Vet. Med..

[72] Spiegel K.A., Havas K.A. (2019). The socioeconomic factors surrounding the initial emergence of peste des petits ruminants in Kenya, Uganda, and Tanzania from 2006 through 2008. Transbound. Emerg. Dis..

[73] Khan S.S., Hossain H., Talukder S., Uddin M.S., Uddin M.A., Siddiqui M.S.I. (2024). A survey on the knowledges, attitudes, behaviours and practices of goat farmers about peste des petits ruminants disease in goats at Haor and bordered areas in Sylhet district of Bangladesh. Vet. Med. Sci..

[74] Kartskhia N., Fischer K., Risatti G., Chenais E., Wennstr P., Chaligava T., Enukidze T., Ståhl K., Vepkhvadze N.G. (2021). Perceptions of pastoralist problems: A participatory study on animal management, disease spectrum and animal health priorities of small ruminant pastoralists in Georgia. Prev. Vet. Med..

[75] Robi D.T., Bogale A., Temteme S., Aleme M., Urge B. (2024). Adoption of veterinary vaccines, determining factors, and barriers in Southwest Ethiopia: Implications for livestock health and disease management strategies. Prev. Vet. Med..

